# A Novel Clinical Nomogram to Predict Transient Symptomatic Associated with Infarction: The ABCD3-SLOPE Score

**DOI:** 10.1155/2021/5597155

**Published:** 2021-04-14

**Authors:** YanQin Lu, QianQian Bi, Wang Fu, LiLi Liu, Yin Zhang, XiaoYu Zhou, Jue Wang

**Affiliations:** ^1^Department of Neurology, Shanghai Tenth People's Hospital, Tongji University School of Medicine, Shanghai, China; ^2^Department of Neurology, Shanghai Hongkou District Jiangwan Hospital, Rehabilitation Hospital Affiliated to Shanghai University of Medicine & Health Sciences, Shanghai, China; ^3^Tongji University School of Medicine, Shanghai, China; ^4^Educational Office, Shanghai Tenth People's Hospital, Tongji University School of Medicine, Shanghai, China

## Abstract

**Background:**

It is hard to differentiate transient symptoms associated with infarction (TSI) from transient ischemic stroke (TIA) without MRI in the early onset. However, they have distinct clinical outcomes and respond differently to therapeutics. Therefore, we aimed to develop a risk prediction model based on the clinical features to identify TSI.

**Methods:**

We enrolled 230 consecutive patients with transient neurologic deficit in the Department of Neurology, Tongji University Affiliated Tenth People's Hospital from March 2014 to October 2019. All the patients were assigned into TIA group (DWI-negative) or TSI group (DWI-positive) based on MRI conducted within five days of onset. We summarized the clinical characteristics of TSI by univariate and multivariate analyses. And then, we developed and validated a nomogram to identify TSI by the logistic regression equation.

**Results:**

Of the 230 patients, 41.3% were diagnosed with TSI. According to the multivariate analysis, four independent risk factors, including smoking history, low-density lipoprotein cholesterol, brain natriuretic peptide precursor, and ABCD3 score, were incorporated into a nomogram. We developed a predictive model named ABCD3-SLOPE. The calibration curve showed good agreement between nomogram prediction and observation. The concordance index (C-index) of the nomogram for TSI prediction was 0.77 (95% confidence interval, 0.70-0.83), and it was well-calibrated.

**Conclusions:**

Smoking history, low-density lipoprotein cholesterol, brain natriuretic peptide precursor, and ABCD3 score were reliable risk factors for TSI. ABCD3-SLOPE was a potential tool to quantify the likelihood of TSI.

## 1. Introduction

A transient episode of neurological dysfunction caused by focal brain, spinal cord, or retinal ischemia, without acute infarction, has been defined as a TIA [1, 2]. With the advent of diffusion-weighted imaging (DWI), it was reported that 29.3-41.5% percent of patients with transient clinical deficit had positive DWI [3–7]. To data, the normative definition for these DWI-positive cases was not yet well-established, while it was termed transient symptoms associated with infarction (TSI) [6]. They are two distinct entities and contribute to different prognoses, even if they share the identical feature that neurological deficits fully recovered within 24 hours of onset. The risk of recurrent ischemic events is much higher in patients with TSI than those with TIA. One study found a rate of recurrent ischemic stroke of 10.8% among TSI patients in 90 days, while 4.3% in TIA [8]. What is more, TSI patients were ten times more likely to suffer from ischemic stroke in 7 days and 4.3 times in 12 months than TIA patients [9, 10]. Therefore, early identification of TSI and aggressive treatment may reduce the risk of recurrent stroke.

The TSI diagnosis is exceptionally dependent on DWI sequence, but the MR image is unavailable at the first one or two days of onset in most medical facilities. Nowadays, there are ample studies of detection and early stroke risk prediction for TIA [8, 11–15], while few studies have investigated TSI's clinical characteristics. For this reason, it is imperative to make investigations on the specific clinical characteristics of TSI and to develop a prediction model derived from clinical features for the identification of TSI.

The purpose of this research is to develop a practical risk prediction tool that can aid in rapidly and accurately triaging patients with high positive predictive value.

## 2. Methods

### 2.1. Patients and Study Design

We collected consecutive 348 patients manifested with transient neurological deficits hospitalized in the Department of Neurology, Shanghai Tenth People's Hospital from March 2014 to October 2019. Patients with neurological dysfunction due to cerebral hemorrhage or other nonvascular causes (hypoglycemia, infection, space-occupying lesions, subdural hematoma, and epidural hematoma) were excluded. Also, patients without a brain MRI were excluded from this study. Two hundred and thirty patients were finally recruited. Brain MRI (Siemens 3.0T Avanto MRI) was required for all study participants. Standardized MRI sequences were necessary per protocol (at least T2-weighted, fluid-attenuated inversion recovery images and DWI sequences). All images were analyzed offline centrally by two experienced readers blinded to clinical and demographic data. The patients were divided into TIA group (DWI-negative) and TSI group (DWI-positive) based on DWI sequence results. We retrospectively analyzed the demographic characteristics, clinical manifestations, risk factors, laboratory examinations, imaging examinations, and other variables.

### 2.2. Grouping Standard

A transient episode of neurological dysfunction caused by focal brain, spinal cord, or retinal ischemia, without acute infarction, was defined as a TIA [1, 2]. Correspondingly, the patient with acute infarction was diagnosed with TSI. According to the definition, all the patients were divided into the TIA group (DWI-negative) and TSI group (DWI-positive) based on the DWI sequence results.

### 2.3. Statistical Analysis

Statistical analysis was performed using IBM SPSS Statistics, Version 22.0 (IBM; Armonk, NY). Categorical variables are expressed as a number or percentage, and continuous variables are expressed as means and SDs. Statistical analysis of categorical variables was done using the chi-square test, while continuous variables were compared using the *t*-test or Mann-Whitney test, as appropriate.

First, a univariate analysis was performed. We used the forward stepwise regression method based on the maximum likelihood ratio to conduct multivariate logistic regression analysis of independent influencing factors in TSI patients. The odds ratio (OR) with 95% confidence intervals (CI) was calculated. *P* < 0.05 indicates that the difference is statistically significant. We then established a risk prediction model and drawn the ROC curve of it and each clinical variable, calculated the sensitivity, specificity, and positive and negative predictive values correspondingly.

We formulated the nomogram based on the multivariate Cox regression model for TSI prediction through the package of rms in R, Version 3.6.3. The prediction performance of the nomogram was measured using the C-index and calibration curves.

## 3. Results

### 3.1. Clinical Features

Of the 230 patients, 41.3% (95 of 230) were diagnosed with TSI. The demographic and clinical characteristics of the patients are listed in Table 1. Compared with the TIA group, there were more male (69.5 versus 51.9%; *P* = 0.007), longer duration of symptoms (30 versus 10 min; *P* = 0.011), higher SBP and DBP on admission (155.14 ± 25.34 versus 144.73 ± 20.65 mmHg and 87.23 ± 12.85 versus 83.45 ± 12.36 mmHg; *P* = 0.001 and 0.026), and higher incidences of unilateral weakness and speech impairment (72.6% versus 50.4% and 38.9% versus 21.5%; *P* = 0.001 and 0.006) in TSI group, whereas there were less memory loss (5.3% versus 14.8%; *P* = 0.03) of atrial fibrillation and smoking, and drinking was higher (12.6% versus 3.0% and 48.4% versus 24.4% and 25.3% versus 14.1%; *P* = 0.007, <0.001, and 0.049).

Biomarker and imaging characteristics are shown in Table 2. It indicated that creatinine (*P* = 0.008), uric acid (*P* = 0.006), total cholesterol (*P* = 0.015), low-density lipoprotein (*P* < 0.001), brain natriuretic peptide precursor (*P* = 0.007), red blood cell (*P* = 0.010), hemoglobin (*P* = 0.023), and hematocrit (*P* = 0.022) in the group TSI are significantly higher than those in the TIA group, and the differences were statistically significant. Similarly, the percentages of patients with severe responsible vessel stenosis or occlusion (23.9% versus 14.0% and 9.1% versus 2.3%; *P* < 0.001) and carotid plaque (76.3% versus 56.0%; *P* = 0.002) were higher than that in the TIA group.  Table 3 showed the multivariable analyses of clinical characteristics of TSI patients, and the smoking history, low-density lipoprotein cholesterol, brain natriuretic peptide precursor, and ABCD3 score are the independent risk factors of TSI.

### 3.2. Model Evaluation

**Figure 1:**
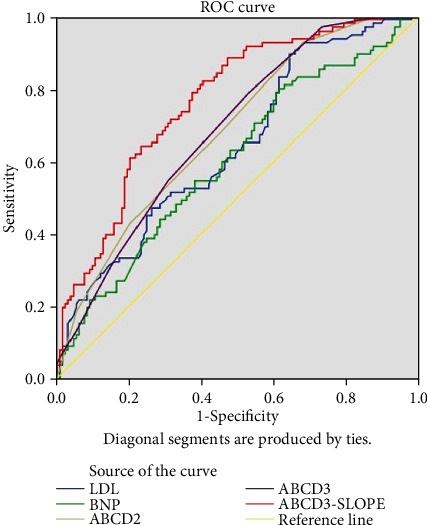
The AUC for the prediction of TSI risk of different models.

Table 4 shows the performance of LDL, NT-pro-BNP, ABCD2 score, ABCD3 score, and our combined risk prediction model. We used the area under the ROC curve to evaluate the diagnosis efficacy (Figure 1). Compared with the other models, the combined model consisting of smoking history, low-density lipoprotein cholesterol, brain natriuretic peptide precursor, and ABCD3 score had the largest area under the ROC curve, whose AUC is 0.762 (95% CI 0.701-0.823), which means it shows better performance.

### 3.3. Construction and Validation of the Nomogram

**Figure 2:**
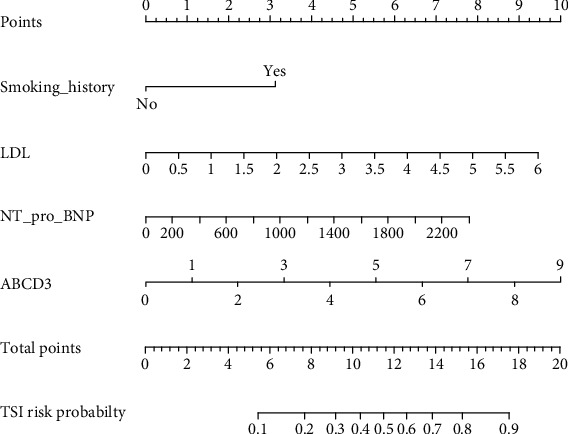
Nomogram for patients with symptoms of TIA. To use the nomogram, the value attributed to a patient is located on each variable axis, and a line is drawn upwards to determine the number of points received for each variable value. The sum of these numbers is located on the total point axis, and a line is then drawn downwards to the probability axis to predict the TSI risk likelihood.

**Figure 3:**
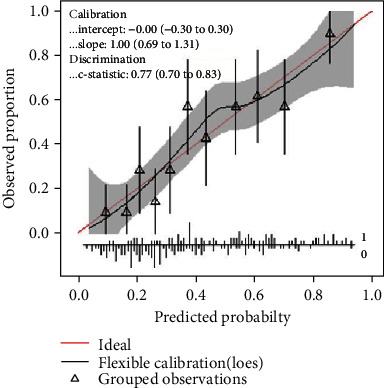
Calibration curve of the combined nomogram. The diagonal red line indicates the ideal prediction by a perfect model.

As the combined model had the best predictive performance, we built a nomogram based on this final model: the ABCD3-SLOPE score (Figure 2). The performance of the nomogram was evaluated by the concordance index (C-index) with 1000 bootstrap resamples, and we measure the discrimination of the model by the area under the ROC curve. A value of 1 indicates perfect discrimination, and 0.5 represents discriminative power equal to randomness. Furthermore, the calibration curve of the combined model (Figure 3) is close to the ideal curve, which demonstrated that it has a well-predictive performance.

## 4. Discussion

Our study suggested 41.3% of the patients with transient neurologic deficits had positive DWI lesions on brain MRI. The rate of TSI in our research was concordant with those in previous studies which reported 29.3-41.5% of the patients with transient symptoms were found acute ischemic lesions by DWI [3–7]. The high incidence of TIS prompted us to develop a practical tool to identify TSI in patients with transient symptoms at the onset of stroke.

Our study found TSI was independently associated with the smoking history, low-density lipoprotein cholesterol, brain natriuretic peptide precursor, and ABCD3 score. We developed the model ABCD3-SLOPE, and it is a practical and convenient tool with the high prediction of TSI in patients with neurological deficits recovered within 24 hours of onset. The score had easy applicability due to the easy and rapid availability of these variables in emergency department at the onset of stroke, and the nomogram indicated the virtue of simplicity and intuition. As demonstrated in Table 4, compared with other models, the ABCD3-SLOPE model had high sensitivity (82.1%) and specificity (59.3%) to identify TSI when the patients presented transient symptoms. The concordance index (C-index) was 0.77 (95% confidence interval, 0.70-0.83) in the internal validation with 1000 bootstrap resamples, and the calibration curve demonstrated that the ABCD3-SLOPE had a well-predictive performance.

TSI is not only an independent entity different from TIA and acute cerebral infarction, but it is associated with a higher risk of recurrent ischemic events as well [4, 16–18]. The patient with a DWI lesion on baseline MRI was 4.3 times (9.1% versus 2.1%; *P* = 0.19) more likely to suffer a subsequent stroke in 1 year than TIA [9]. The 7-day risk of stroke was higher on DWI-positive than DWI-negative patients (1.2% versus 12.3%) [13]. The subsequent risk of ischemic stroke after TIA was 2.4% in 2 days, 3.8% in 7 days, 4.1% in 30 days, and 4.7% in 90 days according to a systematic review and meta-analysis of 206455 unique patients [19]. It is reasonable to assume higher rates of subsequent ischemic stroke in 30 or 90 days in the TSI population. There was a trend that minor stroke patients who received intravenous t-PA had favorable outcomes and the larger proportion of alive and independent defined as Oxfordshire Handicap Score of 0-2 [20]. In the Austrian Stroke Unit Registry, rt-PA-treated patients with mild stroke had a good outcome in comparison to subjects without rt-PA [21]. However, there is no evidence of evidence-based medicine on intravenous t-PA in TIA patients. Thrombolysis is warranted in the TSI population instead of TIA patients probably. Early initiation of existing treatments was associated with an 80% reduction in early recurrent stroke and reduced subsequent hospital bed-days, acute costs, and 6-month disability [22, 23]. Despite the fact that recognition of TSI is of much importance, it remains a challenge for the physician in emergency department, since the TSI diagnosis is exceptionally dependent on the DWI sequence. Chaturvedi et al. reported that no more than 51% of TIA or minor stroke had MR image within two days of initial presentation [24]. In another multicenter study, MRI was performed in 58.5% of patients with TIA or minor stroke [25]. Other validating and simple tools are needed for physicians to distinguish TSI from TIA as traditionally defined.

In our study, we obtained a high global accuracy with the combination of four items that built the model of ABCD3-SLOPE: the smoking history, low-density lipoprotein cholesterol, brain natriuretic peptide precursor, and ABCD3 score. These items were featured with easy and quick availability. It provided a practical risk prediction tool for clinicians in rapid and accurate identification of TSI from TIA population at the earliest when MRI is not available. In consequence, more appropriate and precise treatments could be taken in time.

Some risk factors were found their associations with TSI in recent years. Jia et al. found that dysphasia was associated with acute DWI lesions in patients with transient neurological symptoms [26]. In Crisostomo et al.'s study, patients with positive DWI were 9.6 times more likely to present with symptom duration greater than 1 hour [27], and longer duration of symptoms would yield a higher probability of persistent parenchymal damage that could be detected by MRI. Cigarette smoking has appreciable adverse effects on hemorheological parameters, plasma osmolality, and high fibrinogen level. RBC aggregation in smokers promotes endothelial dysfunction through arterial wall inflammation and the impact of hematocrit on the blood vessel, and the increased blood viscosity may play an adverse role in the dissolution of thrombus [28]. Brain natriuretic peptide precursor is secreted by the cardiac ventricles, increasing serum levels correlate with the severity of left ventricle dysfunction. Left atrial appendage emptying flow velocity (LAAEV) can reflect the left atrial function, and it was independently associated with ischemic stroke in a prospective observational study [29]. As we have known that DOT score [11], Dawson score [12], and ABCD scoring system [30] are clinical diagnostic tools designed for and from TIA population, they fail to be fully applicable to TSI population. The ABCD3-SLOPE model and nomogram is derived from the TSI population. Therefore, it is not only a simple risk assessment tool but of suitability to quantify the likelihood of TSI in the way of visualization.

Although the nomogram model demonstrated reasonable accuracy for the prediction of TSI, there are some limitations. First, the time delay from symptom onset to MRI examination is an essential factor that affects DWI positivity [3], and some patients might be underestimated if the MRI was performed within 24 hours of symptom onset. The time should be classified in the further study. Second, the current study was performed on the basis of a single-center, retrospective database, and it might not have the generalizability of a multicenter trial typically retains. However, we found the baseline characteristics of our patients such as age, gender, risk factors, and clinical symptoms were similar to a large sample size of 1910 patients [31], and we had reason to believe our cohort had a representative of a larger population. Third, although we had well internal validation, we did not have external validation. Hence, a further multicenter, larger validation study is clearly warranted.

## 5. Conclusions

Limb weakness, speech impairment, long duration of symptoms, high ABCD3 scores, and smoking history were the independent risk factors for TSI. The ABCD3-SLOPE and nomogram had a reasonable prediction performance on the recognition of TSI, and we need to design more robust research to explore the best critical ABCD3 scores and symptom duration to predict the risk of TSI and to provide more reliable evidence for other potential predictors.

## Figures and Tables

**Table 1:** Demographic and clinical characteristics of the patients.

Variables	TIA (*N* = 135)	TSI (*N* = 95)	*P* value
*Demographic characteristics*			
Gender (male, %)	70 (51.9)	66 (69.5)	0.007
Age (years)	65 (59.73)	64 (58.71)	0.516
*Clinical data*			
Duration of TIA (min)	10 (3.60)	30 (9.60)	0.011
Frequency in 24 h (%) 1	112 (83.6)	70 (73.7)	0.097
≥2	22 (16.4)	25 (26.3)	
SBPa (mmHg)	144.73 ± 20.65	155.14 ± 25.34	0.001
DBPa (mmHg)	83.45 ± 12.36	87.23 ± 12.85	0.026
*Clinical symptoms*			
Unilateral weakness (%)	68 (50.4)	69 (72.6)	0.001
Numbness or tingling (%)	41 (30.4)	33 (34.7)	0.579
Speech impairment (%)	29 (21.5)	37 (38.9)	0.006
Diplopia (%)	2 (1.5)	0 (0)	0.513
Dizzy (%)	43 (31.9)	22 (23.2)	0.196
Unconsciousness (%)	9 (6.7)	4 (4.2)	0.566
Bilateral amaurosis, hemianopia (%)	11 (8.1)	4 (4.2)	0.286
Memory loss (%)	20 (14.8)	5 (5.3)	0.030
Facial paralysis (%)	7 (5.2)	12 (12.6)	0.053
Torpor (%)	6 (4.4)	4 (4.2)	1.000
Unilateral amaurosis (%)	5 (3.7)	0 (0)	0.079
Weakness of upper limbs (%)	10 (7.4)	3 (3.2)	0.248
*PMH*			
Hypertension (%)	91 (67.4)	64 (67.4)	1.000
Diabetes mellitus (%)	32 (23.7)	26 (27.4)	0.634
Atrial fibrillation (%)	4 (3.0)	12 (12.6)	0.007
Hyperlipidemia (%)	27 (20.0)	19 (20.0)	1.000
Stroke (%)	21 (15.6)	17 (17.9)	0.772
TIA (%)	48 (35.6)	33 (34.7)	1.000
Dual TIAa (%)	28 (20.7)	28 (29.5)	0.173
Coronary heart disease (%)	10 (7.4)	7 (7.4)	1.000
Tumor (%)	6 (4.4)	5 (5.3)	0.776
Renal insufficiency (%)	8 (5.9)	2 (2.1)	0.202
Smoking (%)	33 (24.4)	46 (48.4)	<0.001
Drinking (%)	19 (14.1)	24 (25.3)	0.049
*Drug history*			
Antiplatelet drugs (%)	18 (13.3)	8 (8.4)	0.344
Statins (%)	10 (7.4)	3 (3.2)	0.278
Anticoagulants (%)	1 (1.1)	1 (0.7)	1.000
*ABCD scores*			
ABCD2	3 (2.4)	4 (3.5)	<0.001
ABCD3	4 (2.5)	5 (4.6)	<0.001

PMH: past medical history; SBPa: the first systolic blood pressure after admission; DBPa: the first diastolic blood pressure after admission; dual TIAa prompting medical attention plus at least one other TIA in the preceding 7 days.

**Table 2:** Biomarker and imaging characteristics.

Variables	TIA (*N* = 135)	TSI (*N* = 95)	*P* value
*Biomarkers data*			
CRP (mg/L)	3.02 (3.02, 3.23)	3.02 (3.02, 3.40)	0.306
NSE (ng/ml)	14.27 (12.72, 16.38)	14.18 (12.88, 16.71)	0.484
Folic acid (ng/ml)	7.18 (5.63, 9.63)	7.24 (5.22, 9.68)	0.763
HbA1c (%)	5.85 (5.68, 6.25)	5.90 (5.60, 6.33)	0.700
ALT (U/L)	17.80 (10.80, 25.95)	17.10 (11.00, 23.30)	0.473
CR (*μ*mol/L)	69.50 (59.60, 83.98)	76.90 (66.95, 85.24)	0.008
UA (*μ*mol/L)	317.71 ± 75.57	346.82 ± 80.94	0.006
FBG (mmol/L)	5.10 (4.60, 5.55)	5.10 (4.80, 5.55)	0.195
AST (U/L)	18.00 (15.05, 23.35)	17.20 (14.65, 21.70)	0.283
TC (mmol/L)	4.16 ± 1.11	4.50 ± 0.94	0.015
TG (mmol/L)	1.27 (0.90, 1.65)	1.36 (1.07, 1.72)	0.175
HDL (mmol/L)	1.10 (0.94, 1.33)	1.05 (0.90, 1.25)	0.260
LDL-C (mmol/L)	2.39 ± 0.89	2.83 ± 0.82	<0.001
Calcium (mmol/L)	2.27 (2.22, 2.32)	2.26 (2.21, 2.31)	0.287
Hcy (*μ*mol/L)	11.30 (9.45, 11.89)	11.20 (9.20, 15.15)	0.772
Fib (g/L)	2.59 (2.33, 3.05)	2.56 (2.38, 2.99)	0.837
D-dimer (mg/L)	0.28 (0.22, 0.46)	0.29 (0.22, 0.46)	0.614
NT-pro-BNP (pg/ml)	75.07 (31.04, 138.75)	99.42 (52.76, 239.55)	0.007
WBC (∗109/L)	6.20 (5.24, 7.42)	6.38 (5.34, 7.58)	0.544
RBC (∗1012/L)	4.38 ± 0.54	4.56 ± 0.48	0.010
Hemoglobin (g/L)	133.27 ± 15.94	138.13 ± 15.77	0.023
Hematocrit (%)	40.19 ± 4.58	41.58 ± 4.34	0.022
PTL (∗109/L)	216 (185, 254)	221 (176, 262)	0.853
Neutrophil (%)	3.50 (2.91, 4.55)	3.79 (3.13, 4.56)	0.172
Lymphocyte (%)	1.92 (1.55, 2.40)	1.89 (1.52, 2.20)	0.311
NLR	1.79 (1.36, 2.42)	2.02 (1.51, 2.65)	0.069
MPV (fL)	10.81 ± 0.96	10.80 ± 0.96	0.920
PCT (%)	0.23 (0.19, 0.28)	0.23 (0.20, 0.28)	0.665
PDW (%)	12.70 (11.35, 13.80)	12.70 (11.35, 14.15)	0.678
*Imaging data*			
*Degree of vascular stenosis (%)*			
Mild	99 (76.7)	44 (50.0)	<0.001
Moderate	9 (7.0)	15 (17.0)	
Severe	18 (14.0)	21 (23.9)	
Occlusion	3 (2.3)	8 (9.1)	
*Fazekas score (%)*			
0	31 (23.0)	15 (16.1)	0.136
1	74 (54.8)	52 (55.9)	
2	22 (16.3)	17 (18.3)	
3	8 (5.9)	9 (9.7)	
*Carotid plaque (%)*			
No	59 (44.0)	22 (23.7)	0.002
Yes	75 (56.0)	71 (76.3)	

**Table 3:** The multivariable analyses of clinical characteristics of TSI patients.

Variables	*β*	*P*	OR	95% CI	
				Lower	Upper
Smoking history	1.220	<0.001	3.388	1.265	5.115
LDL	0.404	0.035	1.497	1.081	2.388
NT-pro-BNP	0.003	0.019	1.003	1.001	1.006
ABCD3 score	0.422	<0.001	1.525	1.233	1.853
Constant	-4.058	<0.001	0.017	-	-

**Table 4:** Performance of prediction models.

Predicting factors	AUC	95% CI		Cut-off value	*P* value
		Lower	Upper		
LDL	0.638	0.566	0.710	1.98	<0.001
NT-pro-BNP	0.601	0.527	0.676	48.515	0.009
ABCD2 score	0.681	0.613	0.750	2.5	<0.001
ABCD3 score	0.682	0.614	0.750	3.5	<0.001
Prediction model	0.762	0.701	0.823	0.325	<0.001

## Data Availability

The data are available from the corresponding author on reasonable request.
